# Development and pilot implementation of Iranian Hemolytic Uremic Syndrome Registry

**DOI:** 10.1186/s13023-022-02376-9

**Published:** 2022-06-16

**Authors:** Mina Lazem, Nakysa Hooman, Abbas Sheikhtaheri

**Affiliations:** 1 Department of Health Information Management, School of Health Management and Information Sciences, Iran University of Medical Sciences, Tehran, Iran; 2 Aliasghar Clinical Research Development Center (AACRDC), Aliasghar Children Hospital, Department of Pediatrics, School of Medicine, Iran University of Medical Sciences, Tehran, Iran

**Keywords:** Model, Hemolytic uremic syndrome, Registry, Disease registry, Rare disease, Iran

## Abstract

**Background:**

Patients with Hemolytic Uremic Syndrome (HUS) face late diagnosis and lack of appropriate treatment because of a lack of knowledge and experience in this field. A prerequisite for such knowledge is the development of research infrastructures such as a registry system. Therefore, this study aimed to develop and describe the HUS registry in accordance with the Iranian health system and implement its software system.

**Methods:**

We first interviewed 10 pediatric nephrologists and after analyzing the interviews, we identified the features and requirements and the data related to HUS. Then, during two rounds of the Delphi technique (the first round with 23 participants and the second round with 18 participants), the model of this registry was finalized based on the agreement of at least 75% of specialists. At the next step, based on the agreed requirements, IRI.HUS.Reg (Iranian Hemolytic Uremic Syndrome Registry) software was developed and implemented in a pediatric hospital.

**Results:**

We classified 369 meaning units of interviews in 41 codes and 7 final themes including purposes of the registry (10 codes), inclusion criteria (7 codes), data collection method (4 codes), data quality control (6 codes), data sources (4 codes), data analysis (3 codes) and software features (7 codes). These 7 feature groups (67 subgroups) and 12 data classes (138 data elements) include demographic data, referrals, examinations, clinical signs, causes, laboratory tests, medical histories, paraclinical measures, treatments, outcomes, patient’s status at discharge, and follow-up data were reviewed by the Delphi panelists, and finally, 64 features and 131 data elements were accepted by at least 78% agreement. Then, we developed and implemented a registry software system in a hospital.

**Conclusion:**

We implemented IRI.HUS.Reg based on related features, 12 data classes agreed by specialists, literature review, and comparison with other existing registries. Therefore, the data collected in this registry can be compared with other data from existing registries in other countries.

**Supplementary Information:**

The online version contains supplementary material available at 10.1186/s13023-022-02376-9.

## Background

Patients with Rare Diseases (RDs) face important challenges such as late diagnosis, lack of available treatments, and finding the appropriate health services [1]. Hemolytic Uremic Syndrome (HUS) is also a rare syndrome that often occurs secondary to others diseases. In such cases, it may be difficult to diagnose due to the overlap of HUS and the first disease. This syndrome is one of the thrombotic microangiopathy conditions diagnosed with the three early symptoms of hemolytic anemia along with fragmentocytes, acute renal failure, and low platelet count [2]. There are typical and atypical HUS. Typical HUS usually occurs after a period of diarrhea, mainly caused by pathogens producing Shiga toxin [3] and usually occurs in children under five years [4]. Atypical HUS (aHUS) is often familial and sporadic. The abnormal activation of the complement pathway is one of the key reasons for the physiology of aHUS [3]. In general, HUS is a potentially fatal disease that often occurs in children due to Shiga Toxin-producing Escherichia Coli (STEC) and in some cases, other pathogens [5] which result in a significant effect on the quality of life of patients and mortality [6, 7]. The annual prevalence of HUS is difficult to assess, but its overall prevalence is reported to be two per 100,000 for all age groups and up to six per 100,000 in children under 5 years of age [8]. Furthermore, the annual prevalence of aHUS at all ages was reported 0.23–1.9 per million, and 0.26–0.75 per million in patients 20 years or younger [9]. In Iran, aHUS prevalence is 0.55 per million, and 2.41 per million in children under 15 years old [10]; however, there are no statistics on the prevalence of typical HUS in Iran.

The lack of knowledge and experience related to many rare diseases (such as HUS) is an important health problem. As mentioned in a study, healthcare professionals themselves stated that lack of knowledge was the biggest problem they face when caring for patients with rare diseases and most future physicians do not have basic knowledge about epidemiology, prevalence, and effective care of these patients and urgently need more knowledge and training in this field [11]. A prerequisite for achieving this knowledge is to provide the infrastructure for research initiatives and a better understanding of the distribution and determinants of these diseases, and the development of new treatments and other interventions. This infrastructure can be effectively provided by developing a systematic set of clinical, genetic, and biological data in the patient registry [12]. The aggregation and integration of RD data in a registry are very important because the lack of knowledge and diagnosis of a sufficient number of patients in this type of disease is often due to the lack of integrated data, evidence, and knowledge [13].

Similarly, the development of disease registries for HUS (especially aHUS) is necessary so that with sufficient sampling, natural history and progression and its prognosis in different populations of patients can be evaluated and studied [14]. Previously, we conducted a literature review to identify, investigate and compare the features and data sets of 10 HUS registries. This review indicated and compared the purposes, inclusion criteria, data sources, methods of data collection, and evaluating the quality of data, as well as the scope of the data set of these HUS registries. There are a variety of HUS/aHUS registries in the United States, Italy, France, and Turkey; some of them are also global registries [15]. The purposes of HUS registries often include optimal management and treatment of patients [16, 17], genetic evaluation, estimation of the incidence [14, 18–20], and other epidemiological studies and research [18, 19, 21, 22]. So far, no registry for this syndrome has been set up in Iran. Moreover, Iran does not participate in any of the available registries, including the aHUS global registry [23].

Regarding the importance of the registry in facilitating research and promoting knowledge about the HUS, we conducted this study to provide a model and protocol for implementing a HUS registry for Iran.

## Method

We conducted this study using a mixed-method approach. At first, we conducted a qualitative study (content analysis) to assess the needs and requirements of the registry. This method was previously reported as a useful approach to identify requirements of rare disease registries [24, 25]. Then, we conducted a Delphi study to reach a consensus on the HUS registry requirements. Delphi is a popular method to reach a consensus on disease registries [26, 27]. Finally, we developed and implemented the registry software.

### Phase 1: IRI.HUS.Reg registry requirements

#### Participants

We interviewed pediatric nephrologists and fellows. The sampling method was purposeful. Interviewees were identified by the snowball method and introduced by each participant. The inclusion criteria were having a faculty board and at least one year of working experience in HUS treatment. To expand the diversity of views, specialists working in Ali Asghar, Children's Medical Center, Mofid and Aboozar Children's hospitals (from four Universities of Medical Sciences of Iran, Tehran, Shahid Beheshti, and Ahvaz Jundishapur), as well as the Iranian Society of Pediatric Nephrology were invited to participate. The reason for selecting these hospitals was specialization in pediatric diseases and having a pediatric nephrology department.

The interviews continued until data saturation. Regarding the need for data saturation in qualitative studies, the decision about when data saturation occurs is usually based on the researcher's sense of what he or she hears in interviews [28]. Therefore, it is not possible to determine the number of participants from the beginning. After 10 interviews, the data seemed to be saturated because no new findings emerged. However, we invited two other pediatric nephrologists to be assured regarding data saturation. We also included these two interviews in the final analysis. We invited 17 experts for interviews; however, five of them did not cooperate with us due to their busy schedules.

#### Data collection tools and methods

We developed a semi-structured interview guide with 11 initial questions based on our previous review on HUS registries [15] and other related literature [10, 14, 17, 29–39], and publications regarding the HUS registry [20, 22, 40–43]. Several probing questions were considered for more detailed responses. The content validity of the interview guide was approved by two pediatric nephrologists.

Before each interview, we coordinated the time and place of the interview with the participants and sent the interview questions and consent form to them. To achieve the depth of findings, the first two interviews were conducted as a pilot, and based on their analysis, the writing of some questions became clearer. In this regard, two new questions were added. We asked these new questions in the second session of the interview with these two participants. These two interviews were also considered in the final analysis (Additional file 1).

We conducted the interviews in person at participants' workplaces and in some cases online after prior coordination and appointment. The researcher took notes during the interviews to pursue the concepts in subsequent interviews. After analysis of the responses, if necessary, other questions were asked from the same participants during the next meetings to clarify or complete the responses. Based on the request of some participants, they were interviewed in two sessions. Totally, five participants were interviewed in two sessions. The duration of each interview was between 45 and 65 min.

Rigor is a measure of trustworthiness of qualitative data collection and analysis. Rigor in qualitative research can be roughly compared to reliability and validity in quantitative research. Rigor classic criteria were defined by pioneers of qualitative research, Lincoln and Guba (1985), which include credibility, dependability, conformability, and transferability [44–46].

- For the credibility of the data, the interviews were conducted and analyzed during a period of 4 months (from January to April 2021). A trained and experienced researcher (ML) conducted all the interviews with the guidance of a clinical consultant. This researcher had a history of collaborating in implementing and designing disease registries. Furthermore, a pediatric nephrologist with experience in the rare disease registries and a researcher with 15 years of experience in designing registry programs participated in the data analysis. To obtain diverse and in-depth responses, we sampled specialists from two metropolises, four different universities, and the Iranian Society of Pediatric Nephrology with the research team consensus. During the interviews, several discussion and consultation meetings were held with the presence of registry specialists and pediatric nephrologists to comment on the emerged themes.
- For the dependability of the data, we used audio recording and taking notes of the participants’ statements as well as their non-verbal communications to record the expressions and perspectives of the interviewees. The two researchers transcribed the interviews separately and simultaneously, matched the transcriptions with the audio files in a joint session. Also, transcripts were returned to participants for any comments and feedback. They confirmed their answers and, in some cases, cleared up any ambiguities.
- To ensure confirmability, we discussed the themes obtained by each researcher in regular weekly meetings. In this way, we reached a consensus on these findings.
- For data transferability, we reported the content of the interview guide, the number of interviewees, and their personal, academic, and professional data, as well as their quotes.

#### Data analysis

We transcribed the verbal responses and quotes recorded during interviews as well as non-verbal communication perceived by the researcher immediately after the end of each interview word by word. Transcribed texts were entered into MAXQDA software and analyzed by using the content analysis method. Accordingly, two authors identified and coded the meaning units in quotes based on the framework obtained from the existing literature [15]. At the next step, they placed the conceptually relevant and similar codes in the sub-categories. The 32 key criteria for critical evaluation of qualitative research [47] for this phase of research are presented in Additional file 2.

### Phase 2: Developing IRI.HUS.Reg model using a Delphi study

#### Participants

By developing a Delphi study, we attempted to reach a consensus between specialists and provided our registry model. The sampling was purposeful with criteria similar to the previous phase, as well as the participants’ agreement to participate in the study. In this regard, among the specialists working in the four mentioned hospitals and the Iranian Society of Pediatric Nephrology, the questionnaire was sent to 30 specialists in the first round, including the nephrologists who participated in the interview, and 23 specialists responded after two reminders. In the second round, 18 specialists participated and 5 specialists refused to cooperate due to their busy schedules.

#### Data collection tools and methods

According to the literature review, comparison of HUS registries [15], and the results of the previous phase, we developed a Delphi questionnaire with two parts: 1) features of registry (67 questions) and 2) related data classes and elements (138 questions) on a five-option Likert scale (1: strongly disagree, 2: disagree, 3: neutral, 4: agree and 5: strongly agree). The face and content validity of this questionnaire was investigated and confirmed by two pediatric nephrologists. They did not participate in the interview and Delphi study.

In the first round, the feedback received from 23 participants was analyzed and the results were returned to them in the second round. The second round Delphi questionnaire was similar to the first-round questionnaire and consisted of two parts of 1) features (5 questions) and 2) data elements (10 questions) (between 25 and 75% agreement) along with the mean score of each question obtained at the first round of Delphi. Finally, by analyzing the feedback of the second round of the Delphi study, the HUS registry model was finalized.

Our private IT team designed the registry software in several steps with the cooperation of a pediatric nephrologist. We also conducted a usability evaluation with the participation of four pediatric nephrologists using the Questionnaire for User Interface Satisfaction (QUIS). This questionnaire has 27 questions in five dimensions, each of which is measured on a scale of 10-point scale (0 to 9) [48]. They had access to the software and after two weeks, they were asked to complete the questionnaire.

#### Data analysis

We used descriptive statistics to analyze data. The criterion for the approval of each of the features and data elements was its acceptance (consensus on options 4 and 5 of Likert scale), by at least 75% of the specialists.

The feature or data elements with at least 75% consensus on options 1 and 2 of the Likert scale, were also excluded from the model. Those were between 25 and 75% of the acceptance were reconsidered at the second round of the Delphi. Specialists reached a consensus on all features and data elements in two rounds and no more rounds were needed. For software usability evaluation, we calculated the mean scores of the QUIS questionnaire using SPSS version 22. Mean scores were considered at three levels: poor (0–3), medium (3.1–6), and acceptable usability (6.1–9).

## Results

### Qualitative study

#### Profile of participants

According to Table 1, phase 1 participants included 12 pediatric nephrologists with the same number of gender (n = 6, 50% each). All of them had more than or equal to five years of working experience in HUS diagnosis and treatment and often specialized in pediatric nephrology (n = 11, 92%), and one was a fellow in this field.

**Table 1:** Themes and sub-themes regarding to the IRI.HUS.Reg model

Theme	Sub-themes	No. of interviewees
1. Purposes of implementing the IRI.HUS registry	Assisting in HUS-related clinical research	7
	Accessing to patients' information in their various referrals	6
	Identifying the underlying causes of HUS (to achieve related treatment)	6
	Improving the diagnosis/treatment of HUS	6
	Increasing medical knowledge for HUS management	4
	Discovering the infectious sources that cause HUS	3
	Supporting health policy for drug import	3
	Determining the prevalence of HUS in Iran	3
	Enabling international participation in the field of HUS	2
	Organizing a campaign for patients to receive any supports	1
2. Inclusion criteria	Uremia and reduced renal function	9
	Microangiopathic hemolytic anemia	9
	Reduced platelet count	8
	Increased lactic dehydrogenase	5
	Negative Coombs test	3
	Diagnosis of HUS by a nephrologist	2
	Only patients under-five years	2
3. Data collection	Data collection by the patient's physician	5
	Using trained/expert people to collect data	5
	Case finding through HUS diagnostic ICD code in medical records	4
	Centralized data collection by a reference center	2
4. Data quality control	An expert or trained person for data quality control	5
	Entering the data of each patient by his/her physician	4
	Continuous evaluation of data quality	4
	Random verification of data by an expert	3
	Quality control feedback to data collectors	2
	Evaluation of data quality by the Iranian Society of Pediatric Nephrology	1
5. Data sources	Hospitals (especially pediatric referral centers)	10
	Outpatient centers (especially related to follow-up or pre-hospital information)	7
	Specialized laboratories (nephrology or genetics)	2
	Urban or rural health centers	2
6. Analysis of registry data	Determining the ratio (percentage) of patients in terms of each variable	7
	Determining the average values of each variable	7
	Comparative charts of patient data	4
7. Registry software specifications	Optimal outputs	3
	Possibility of modifying and editing data	2
	Software alerts	2
	Patient follow-up reminder	2
	No need for additional typing	2
	Automated calculations	2
	Enabling to upload patient reports to software	2

*HUS* Hemolytic Uremic Syndrome; *ICD* International Classification of Diseases; *IRI.HUS.Reg* Iranian Hemolytic Uremic Syndrome Registry

We extracted 369 meaning units from interviews and organized them into 41 unique codes and 7 final themes including 1- purposes of the HUS registry (10 codes), 2- inclusion criteria (7 codes), 3- data collection method (4 codes), 4- data quality control (6 codes), 5- data sources (4 codes), 6- data analysis (3 codes) and 7- registry software features (7 codes). The final themes along with the relevant sub-themes are reported in Table 1.

Below are some quotes on themes:

1. Purposes of the HUS registry

One of the important purposes of HUS registries was to assist in clinical research and access patients' information in their various referrals:

“If there is HUS registry, then if a group studies HUS, they will normally have all the data needed for the study in an optimal and centralized way.”(P3)

“Another good thing that can be said for this registry is the maintenance of patient data in a centralized system for further follow-up and treatment of HUS patients.”(P2)

2. Patient inclusion criteria for HUS registry

The most important inclusion criteria were HUS-specific triad including reduced renal activity (increased creatinine and Blood Urea Nitrogen (BUN)), microangiopathic hemolytic anemia, and reduced platelet count in these patients.

"The symptoms of uremia, creatinine raise, and BUN in these cases indicate renal dysfunction that is among the definitive inclusion criteria of this syndrome."(P10)

"Another important inclusion criterion is microangiopathic thrombotic anemia, which is a hemolytic type."(P7)

3. Data collection method in HUS registry

The specialists stated that the most appropriate persons for collecting data were nephrologists treating patients.

"The person in charge of collecting HUS data should preferably be treating physician."(P2)

They referred to different types of important data elements for data collection, which is shown in Table 2.

**Table 2:** The data classes and elements proposed by the participants for the IRI.HUS.Reg

Data classes	Data elements
Demographic	Name, last name, father's name, Identification (ID) code, age, gender, address, phone
Physical examinations	Edema or dehydration of the patient's body and heart/lung sound auscultation Blood pressure, vital signs, height, weight, Body Mass Index (BMI)
Signs and symptoms	Diarrhea, vomiting, fever, nausea, paleness, fatigue, weakness, lethargy, high blood pressure, discoloration of the urine (blood in the urine) and decreased urine volume (with duration), swelling in the organs
Underlying disease and risk factors (causes)	Medications taking, radiotherapy or chemotherapy, diet Exposure to contaminated sources (such as contaminated water and food) Patient living area (villages, suburbs, contaminated areas with unfavorable sewage status, etc.) Dealing with pesticides or livestock Scorpion bites (like Gadim scorpion) Underlying diseases such as cancer, hypertension, collagen vascular diseases, deficiency of some immune factors or complement components, genetic diseases, cardiovascular diseases
Past and family history	Age at onset of symptoms History of gastrointestinal and respiratory infections (especially Shiga toxin infection) in the previous two weeks (bloody diarrhea or cold and acute pediatric pneumonia) Past history of HUS in the patient or patient's family (sibling with previous HUS)
Laboratory data	Hemoglobin, platelet count, Peripheral Blood Smear (PBS) (to check for Shistocyte), creatinine and urea reticulocytes, lactic dehydrogenase, calcium, sodium, potassium phosphorus, urine output, presence of red and white blood cells in the urine, cast in urine, stool culture, coombs, complement-related factors such as C3, C4, CFH CFI and CHB, Cluster of Differentiation 46 (CD46), A Disintegrin and Metalloproteinase with a Tthrombospondin type 1 motif, member 13 (ADAMTS13), Antinuclear Antibody (ANA), liver tests such as Serum Glutamic-Pyruvic Transaminase (SGPT) and Alanine Aminotransferase (ALT), albumin and Protein Total (PT)
Paraclinical measures	Ultrasound of the abdomen and other organs of the body, echocardiography, and MRI
Treatment, medications	Supportive therapies, medications such as antibiotics, antihypertensives such as Enalapril, Anticoagulant, Eculizumab (dosage, prescription method, and duration of use), Immunosuppressant drugs, Methylprednisolone pulse therapy, Monoclonal antibodies, Plasmapheresis, Plasma exchange (plasma rate and duration of treatment), Hemodialysis (HD) or Peritoneal Dialysis (PD) (with results) and kidney transplantation (with results)
Complications and outcomes	Recurrence of HUS (especially atypical type), severe renal failure, severe anemia, neurological complications such as seizures, diabetes, hypertension, and increased blood protein levels
Patient’s status at discharge	Recovery, along with renal failure, recommend to follow up, and Against Medical Advice (AMA)
Patient follow-ups	Evaluation of clinical signs, laboratory findings, medications, ultrasounds, and other paraclinical procedures every few months (once every three to six months)

*CFH* Complement Factor H

*CFI* Complement Factor I

*CHB* Complement Factor B

*C3* and *C4* Most frequently proteins of the Complement system

*IRI.HUS.Reg* Iranian Hemolytic Uremic Syndrome Registry

*MRI* Magnetic resonance imaging

4. Data quality control in HUS registry

Hiring a nephrologist or a trained person for data quality control, and continuous evaluations of the data quality were considered the most effective ways to increase data quality.

“A trained specialist should review and control the collected data, verify the diagnostic or accuracy of this data and then entered it.”(P1)

“After all, the most important solution in my opinion for collecting high-quality data is continuous data monitoring.”(P8)

5. HUS registry data sources

Due to HUS treatment with inpatient care, specialized hospitals (especially specialized children's hospitals) were mentioned as the main sources of data.

“Information for patients with HUS is usually available in pediatric specialized centers, especially in specialized children's hospitals, or pediatric nephrology wards in hospitals.”(P7)

6. Analysis of HUS registry data

Most of the requested data analyses registry data were related to determining the average of recorded values.

“Averaging is usually used to analyze the data in general because until we do not know the average increase in BUN/creatinine or the number of people infected with E. coli (Escherichia coli) in different cities, we cannot make an accurate decision about the disease.”(P1)

7. Features of HUS registry software

The most important features were the optimal outputs, the possibility of editing the data, and a reminder for patient follow-up.

“The registry system should have optimal output, for example, we can transfer our output to Excel.”(P7)

“In the registry, because usually a lot of data should be entered, it should be editable and it is possible to correct the data until the fields are complete.”(P6)

“Registry software should be able to remind the physician of periodic visits and tests.”(P1)

### Delphi study

The number of specialists participating in the Delphi technique was 23 and 18 in the first and second rounds.

In the first and second rounds, 61% and 67% of them were female. All of them were specialists in pediatric nephrology and had more than or equal to five years of working experience in HUS treatment.

The results of the first round, based on seven groups of registry features (67 subgroups) and 12 classes of related data (138 data elements) are reported in Tables 3 and 4, respectively.

**Table 3:** Results of the first round of the Delphi regarding features of the IRI.HUS.Reg

Features		Percentage of agreement (options 4 and 5)
Purposes of implementing the IRI.HUS registry	Promoting HUS research	100
	Accessing to patients' information in their various referrals	100
	Identifying the underlying causes of HUS	100
	Improving the diagnosis/treatment of HUS	100
	Increasing medical knowledge for HUS management	83
	Discovering the infectious sources that cause HUS	87
	Supporting health policy for drug import	91
	Determining the prevalence of HUS in Iran	100
	Enabling international participation in the field of HUS	100
	Organizing a campaign for patients to receive any supports	100
Inclusion criteria	Uremia and reduced renal function	100
	Microangiopathic hemolytic anemia	100
	Reduced platelet count	100
	Increased lactic dehydrogenase	100
	Negative Coombs test*	44
	Diagnosis of HUS by a nephrologist	100
	Only patients under-five years*	44
	Typical HUS	96
	Atypical HUS	96
Data collection	Retrospective	100
	Prospective	100
	Data collection by the patient's physician	100
	Using trained/expert people to collect data	100
	Case finding through HUS diagnostic ICD code in medical records*	74
	Centralized data collection by a reference center	100
Data quality control	An expert or trained person for data quality control	100
	Entering the data of each patient by his/her physician	100
	Continuous evaluation of data quality	100
	Random verification of data by an expert	100
	Determining mandatory fields for data collection	100
	Quality control feedback to data collectors	100
	Evaluation of data quality by the Iranian Society of Pediatric Nephrology	78
	Following data quality guidelines	100
	Using of data quality indicators	100
	Training courses on data quality	100
	Continuous monitoring of incomplete data	100
	Data quality control at the moment it enters the registry (auto by software)	100
Data sources	Hospitals (especially pediatric referral centers)	100
	Pediatric nephrologist offices	100
	Specialized clinics	100
	Specialized laboratories (nephrology or genetics)*	52
	Urban or rural health centers⁑	13
	Research centers	100
Analysis of registry data	Determining the ratio (percentage) of patients in terms of each variable	100
	Determining the average values ​​of each variable	100
	Determining the ratio of variable values to each other (e.g. the ratio of patients number under five to over five years)*	44
	Comparative charts in terms of each variable	100
Registry software specifications	Software quality control when entering data (such as software alert and warning about the admission date before the discharge date)	100
	No need for additional typing	100
	Automatic calculations when entering data (such as automatic calculation of BSA based on patient height and weight)	100
	Enabling to upload patient reports to software (such as genetic test reports)	100
	Displaying the status of completing or not completing the patient questionnaire in the main list of patients	100
	Possibility of modifying and editing dataا	100
	Displaying the range of normal values of laboratory variables	87
	Displaying a reminder of the date to follow-up patients in the main list of patients	96
	Displaying follow up termination date in the main list of patients	100
	Displaying questionnaires editing date in the patient list	100
	Using a unique patient identification ID	91
	Non displaying patients of the other users in the patient list of physicians	96
	Enabling to receive username and password for new users	100
	Excel spreadsheet outputs	100
	PDF outputs	100
	Chart outputs in three shapes: pie, bar, and linear	100
	Possibility of default and custom reports	100
	Possibility to search for patients based on demographic variables for the user	100
	Possibility to register specialists of different hospitals in the registry as a participant	100
	Possibility to correspond with the registry admin for participants (via email)	100

*BSA* Body Surface Area

*HUS* Hemolytic Uremic Syndrome

*ICD* International Classification of Diseases

*ID* Identification

*IRI.HUS.Reg* Iranian Hemolytic Uremic Syndrome Registry

*PDF* Portable Document Format

^*^Reassessed in the 2nd round

⁑Rejected in the first round

**Table 4:** Classification of data classes and elements of the IRI.HUS.Reg in the first round of the Delphi technique

Data classes	Accepted	Rejected	Delphi 2nd round
Demographic	Name*, Family name*, Father's name*, ID Code*, Date of birth*, Age*, Gender*, Ethnic, Address, Place of birth, Phone	–	–
Referral and hospitalization	Hospital name*, Patient referral center (hospital, office, clinic)	Patient referring physician	–
Physical examinations	Observation of edema* or dehydration*, Heart/lung sound auscultation, Blood pressure*, Vital signs, Height*, Weight*, Body Mass Index (BMI)	–	–
Signs and symptoms	Diarrhea* (bloody, non-bloody), Vomiting*, Nausea*, Abdominal pain*, Fever*, Paleness, Fatigue, Weakness and lethargy, Headache, Vertigo, Increased blood pressure*, Urine discoloration* (blood in the urine), Decreased urine volume*, Swelling in the limbs	–	–
Underlying disease and risk factors (causes)	Exposing to contaminated sources (such as contaminated water and food), Patient living area (villages, suburbs, etc.), Medications taking, Lack of complement components*, Underlying genetic diseases*, Cancer or malignancy, Underlying collagen vascular diseases, Underlying heart disease, Underlying kidney disease*, Underlying high blood pressure, Underlying infections, Underlying systemic diseases	–	Exposing to radiotherapy or chemotherapy, Diet, Contact with livestock, Contact with agricultural pesticides, Scorpion bites (like Gadim scorpions)
Laboratory tests	Hematology, Urine analysis*, Urine culture, Stool culture, Biochemistry, Immunology, Hemoglobin*, Platelet count*, Peripheral blood smear*, Creatinine* and urea*, Reticulocytes, Lactic dehydrogenase*, Calcium, Sodium, Potassium, Phosphorus, Urine output*, Presence of RBC and WBC in urine, Cast in urine, Coomb, Factors related to the complement system such as C4, C3, CFB, CFI, CFH, ADAMTS13, Antinuclear Antibody (ANA, Albumin protein, Total protein, Genetic test result, and upload report	–	–
Disease history	Past history*, Family history (sibling)*, Age at onset of symptoms*, Number of days of onset of symptoms at admission, Duration of diagnosis until patient registration, History of gastrointestinal infection (especially Shiga-toxin) in the previous two weeks (dysentery), History of respiratory infection two weeks ago (cold or acute pneumonia), History of systemic diseases, History of kidney transplantation	–	Parental consanguineous marriage
Paraclinical measures	Abdominal ultrasound and its outcome, Echocardiography and its outcome, MRI and its result, Chest photo and its result, Brain CT (Computerized Tomography) scan and its result	–	–
Treatments and medications	Supportive therapies, Antibiotics, Lowering blood pressure like Enalapril, Anticoagulants, Coagulation drugs, Eculizumab, Rituximab, Immunoglobulins, Steroids, Immunosuppressive drugs, Pulse methylprednisolone, Monoclonal antibodies, Dosage of drugs, Duration of medication, Date of consumption of the first dose, Plasma injection*, Plasma exchange*, Hemodialysis or peritoneal dialysis* (with results), Kidney transplant (with results), Splenectomy (with results), Liver transplantation* (with results)	–	Date of the first treatment
Complications and outcomes	Recurrence of the disease*, Severe kidney failure*, Severe anemia, Neurological complications such as seizures, Diabetes, Hypertension, Increased blood protein levels in the long term, Side effects of medications and treatments (during hospitalization), The severity of complications and outcomes, Depression, Bleeding, Sepsis, Other serious infections, Death* (date and main cause of death)	–	Systemic lupus erythematosus, Meningococcal infections
Patient’s status at discharge	Recovery, Discharge along with renal failure, Recommend to follow*	–	Against Medical, Advice (AMA)
Patient follow-up	Clinical signs, Laboratory findings, Medications used, Paraclinical procedures performed	–	–

*ADAMTS13* A Disintegrin and Metalloproteinase with a Tthrombospondin type 1 motif, member 13

*CFH* Complement Factor H

*CFI* Complement Factor I

*CHB* Complement Factor B

*C3 and C4* Most frequently proteins of the Complement system

*HUS* Hemolytic Uremic Syndrome

*ID* Identification

*IRI.HUS.Reg* Iranian Hemolytic Uremic Syndrome Registry

*MRI* Magnetic Resonance Imaging

*RBC* Red Blood Cell

*WBC* White Blood Cell

^*^Mandatory data elements

Table 3 shows that "urban or rural health centers" were rejected. "Negative Coombs test", "only patient under-five years", "case finding through HUS diagnostic ICD (International Classification of Diseases) code", "specialized laboratories", and "determining the ratio of variable values to each other" were re-assessed in the second round of Delphi.

Table 4 shows that in the first round of Delphi, only "patient referring physician" was rejected and 10 data elements were re-assessed in the second round (details of the results of the first round of Delphi are given in Additional file 3, Table 1). The results of the second round of Delphi are presented in Table 5.

**Table 5:** Results of the second round of Delphi regarding features and data elements of IRI.HUS.Reg

Row	Features and data elements of the registry model		Percentage of agreement (options 4 and 5)
1	Features	Negative Coombs test	100
2		Only patients under-five years*	11
3		Case finding through HUS diagnostic ICD code in medical records	100
4		Specialized laboratories (nephrology or genetics)	100
5		Determining the ratio of variables values to each other (e.g. the ratio of patients number under five to over five years)*	6
1	Data elements	Exposing to radiotherapy or chemotherapy	100
2		Contact with livestock	78
3		Diet*	6
4		Contact with agricultural pesticides*	0
5		Scorpion bites (like Gadim scorpions)	100
6		Parental consanguineous marriage*	11
7		Date of the first treatment*	17
8		Systemic lupus erythematosus*	6
9		Meningococcal infections	100
10		Against Medical Advice (AMA) *	0

*ICD* International Classification of Diseases

*Finally rejected

According to Table 5, “only patient under-five years" and "determining the ratio of variable values to each other" were rejected. Six data elements including "diet", "contact with agricultural pesticides", "parental consanguineous marriage", "date of first treatment", "systemic lupus erythematosus" and "discharge Against Medical Advice (AMA)" were also rejected, and other data elements were collectively agreed upon.

### IRI.HUS.Reg software implementation

The final model of IRI.HUS.Reg consisted of seven feature groups (64 final features) and 12 data classes (131 data elements).

Based on this model, our private IT team developed and implemented HUS registry software using C# programming language, ASP.NET, and SQL as the database. Based on users' additional feedback on words, terms, messages, and how to organize registry application screens, software modifications were finalized by our IT team. The level of user satisfaction with the usability of the software in different dimensions of QUIS was at an acceptable level (Additional file 4, Table 1).

**Fig. 1:**
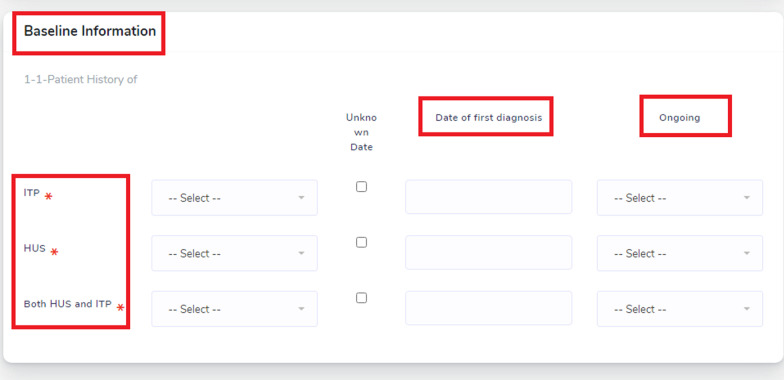
Patient history-related data in IRI.HUS.Reg

The users (nephrologists) can access the home page online by entering the “http://hus-iran.iums.ac.ir”. The Registration section is for registering new professionals or researchers from different health centers who intend to participate in the registry. Figure 1 shows the patient's history in terms of the history of Immune Thrombocytopenic Purpura (ITP), HUS, or both in the past, and whether these conditions were first diagnosed, continued, or not.

The reports of the registry are also in the form of individual default reports (with the possibility of selecting and combining different variables). The reports can be downloaded in two formats including Excel and PDF. In addition to the reports, comparative diagrams of the performance of the participants can also be extracted from this registry software.

## Discussion

According to pediatric nephrologists, we designed the registry model and implementation protocol including seven features including purposes, inclusion criteria, data collection method, data quality control, data sources, data analysis as well as registry software specifications. This registry is implemented centrally in Ali Asghar Children's Hospital as the hub of the registry and is currently at the pilot phase.

1. IRI.HUS registry governance

Currently, the IRI.HUS.Reg steering committee consists of four pediatric nephrologists, two health information management specialists, and one software developer. We identified different stakeholders including specialized pediatric hospitals in the country, each of which will be contacted to sign a data sharing and cooperation agreement. Iranian Society of Pediatric Nephrology was also informed about this registry and will have representatives in the steering committee. Some members of this society were involved in this study to develop this registry model. Furthermore, this society will notify pediatric nephrologists of the purposes of the IRI.HUS.Reg to promote this registry. In Iran, vice-chancellors of research in the ministry of health and medical universities are responsible to supervise and evaluate disease registries. We informed the ministry of health about this registry for their scientific and possibly financial support. This registry is implemented under the governance of the Iran University of Medical Sciences. The vice-chancellor of research in this university was also informed and approved the proposal of this registry. Therefore, this university funded this registry and provided servers to host the registry software.

2. Purposes of IRI.HUS registry

IRI.HUS.Reg aims to help researchers with various clinical and epidemiological studies on HUSand clinical evaluations such as the effectiveness of Eculizumab. Given that our country is also composed of different ethnicities [49], by establishing the HUS registry, different underlying causes can be addressed and therapeutic plans related to each cause can be investigated in different ethnicities.

Another purpose of our registry is to use the data in patients' continuous visits to follow up outcomes. This registry is online and participating nephrologists can access it with mobile internet to access his/her patients’ data. We think that online access to patients’ information at any time and access to the data for research motivates nephrologists to participate in this registry.

Other HUS registries consider increased knowledge about the disease history, evaluation of clinical treatments, drug safety, and quality of care [39], and increased research opportunities [18, 33, 50] as their purposes which are consistent with our purposes.

3. Inclusion criteria for IRI.HUS registry

The inclusion criteria in IRI.HUS.Reg includes both types of HUS (typical and atypical) with the specific triad of acute renal failure (with laboratory-approved uremia), microangiopathic hemolytic anemia, and reduced platelet count. These criteria have been specifically reported in scientific and professional references [51–53] for the definitive diagnosis of HUS. Additionally, the diagnosis of HUS by a pediatric nephrologist is an important inclusion criterion.

Woodward et al. [39] introduced inclusion criteria such as patients of all ages and clinical diagnosis of atypical HUS (with laboratory approval of the absence of Shiga toxin). They excluded patients with a complement gene mutation or factor H antibody. In the study by Soraru et al. [37], inclusion criteria are patients with Thrombotic Thrombocytopenic Purpura (TTP); and HUS patients with Shiga toxin origin are excluded. Therefore, in our registry, such as these studies, patients with typical or atypical types of this syndrome without age limitation will be included.

4. IRI.HUS registry data sources

The main data source of in IRI.HUS.Reg is the specialized pediatric hospitals because these patients in Iran, are mainly managed in hospitals. However, in our registry, some data such as HUS history, specialist counseling, and patient follow-up data will be obtained from nephrologists' offices and outpatient clinics. Although data collection from these outpatient centers is difficult, we are planning to sign a cooperation agreement with them to involve them in research outputs and publications. In addition, we think that access to their patients’ data may be helpful in this respect.

5. Data and data collection method in IRI.HUS registry

It is necessary to collect both types of data related to patients diagnosed before the implementation of the registry (retrospectively) and also the data for future follow-ups [54] (prospectively). In our registry, as the registries mentioned in [35–37], the data will be collected from pediatric hospitals, nephrologists' offices, and outpatient clinics retrospectively and prospectively. On contrary, some HUS registries only collect data retrospectively [32] or prospectively [39].

IRI.HUS.Reg will collect 131 data elements in 12 data classes. These data elements cover the most information needed to research this rare syndrome. George et al. [30, 55] and Metjian et al. [33] in their studies pointed to data classes similar to our registry. Although 131 data elements may result in a burden in workflow, we are planning to connect this registry to hospital information systems and laboratory information systems to capture demographic and laboratory data directly. In addition, only 43 data elements are mandatory.

6. Data quality control in IRI.HUS registry

In our registry, participating pediatric nephrologists will participate in data collection and improve the quality of their data based on the feedback received from the software and the principal investigator (PI) of the registry. The PI of the registry (a pediatric nephrologist) will routinely control the received data and give feedback to participants. We are also planning to conduct yearly data quality studies with the participation of involved nephrologists to evaluate randomly selected registered cases by comparing the registered data with the actual patients’ data in their medical records. Licht et al. [56] and Woodward et al. [39] pointed to the increased participation of specialists in collecting, editing, and correcting data-related defects. Other HUS registries [21, 22] conduct a monthly evaluation of the quality of HUS data. Therefore, the solutions of data quality control in our registry are similar to other registries.

7. Data analysis plans in IRI.HUS registry

In our registry, analytical reports are based on the raw values of patients' data, the average of numerical values, and the ratio (percentage) of the number of patients in terms of qualitative variables. Other HUS registries provided their results and reports using descriptive statistics such as average and median on quantitative variables (such as age at the time of diagnosis) and percentage of patients on qualitative variables [14, 32, 35, 56]. To this end, we will prepare and distribute these reports annually by collecting any number of patients.

8. IRI.HUS Registry software

We found that nephrologists preferred software with optimal outputs, alerts, the possibility of modifying and editing data, a reminder for patients’ follow-up, no need for additional typing, automatic calculations of some variables, and the possibility to easily download patient reports that we considered in the design of our software. Also, another important specification of our registry software is the possibility to export optimal outputs in Excel format. In case of feedback from the quality control officer regarding the non-approved data, correction of data errors and defects after the entry is also possible.

### Limitations

We had limited access to a sufficient number of pediatric nephrologists to participate in the interview and Delphi study. They were from four universities in two cities; therefore, the results may not reflect all pediatric nephrologists’ opinions. Another limitation was that the registry steering committee was only for the pilot implementation of the registry. Establishing the final committee requires the presence of representatives from the Iranian Society of Pediatric Nephrology and hospitals and possibly patients.

Another limitation was the organization of the variables and determination of the minimum mandatory data items. Most of the data items were mandatory at first, but our pilot showed that many of them might not be completed. Furthermore, the organization of variables in different questionnaires was not approved by experts in some cases. According to previous studies [57, 58], other registries also faced such limitations and this could cause problems in a registry. Therefore, during the pilot, by holding various meetings, the minimum mandatory data and their organization were finalized with the participation of nephrologists.

Another possible limitation is to motivate nephrologists to participate in the registry and to continue it in the future. In this regard, it is necessary for the registry steering committee to determine the necessary benefits to create incentives, including participation in publications. Obtaining ethical approvals from research centers and universities at the local levels for the participation of nephrologists across the country is another limitation, for which a clear policy should be set in the future.

Evaluating data quality, giving feedback and correcting data is time consuming. Other studies have reported similar problems related to data quality evaluation [57, 58]. In this case, hiring a qualified person for these functions will be required. Finally, our registry software was designed in English. Some nephrologists emphasized that it was better to design bilingual (English-Persian) software, which can be addressed in the future to overcome this limitation.

## Conclusion

IRI.HUS.Reg collects data regarding 12 data classes and 131 data elements. This registry is designed based on the specifications considered by pediatric nephrologists, literature review, and comparing existing registries, so collecting data in this registry allows the data to be comparable with other HUS registries in the world.

## Supplementary Information

**Additional file 1**: Interview questions.

**Additional file 2**: Report form of consolidated criteria for reporting qualitative studies (COREQ).

**Additional file 3**: Results of the first round of Delphi technique regarding data classes and elements.

**Additional file 4**: Results of the software usability evaluation.

## Data Availability

Data sharing is not applicable to this article as no datasets were generated or analyzed during the current study.

## Abbreviations

ADAMTS13: A Disintegrin and Metalloproteinase with a Tthrombospondin type 1 motif, member 13
aHUS: Atypical HUS
ALT: Alanine Aminotransferase
AMA: Against Medical Advice
ANA: Antinuclear Antibody
ASP.NET: Active Server Pages.Network
BMI: Body Mass Index
BSA: Body surface area
BUN: Blood urea nitrigen
CBC: Cell blood count
CFH: Complement Factor H
CFI: Complement Factor I
CHB: Complement Factor B
CT: Computerized tomography
C3 and C4: Most frequently proteins of the complement system
C#: A general-purpose, procedural computer programming language
CKD: Chronic KIDNEY DISEASE
E. coli: Escherichia coli
HD: Hemodialysis
HUS: Hemolytic Uremic Syndrome
ICD: International Classification of Diseases
ID: Identification
ITP: Immune Thrombocytopenic Purpura
IRI.HUS.Reg: Iranian Hemolytic Uremic Syndrome Registry
MVC: Model-view-controller
MRI: Magnetic resonance imaging
PBS: Peripheral blood smear
PD: Peritoneal dialysis
PDF: Portable document format
PT: Protein total
QUIS: Questionnaire for user interface satisfaction
RBC: Red blood cell
RDs: Rare Diseases Registries
SGPT: Serum Glutamic-pyruvic Transaminase
SQL: Structured Query Language
STEC: Shiga Toxin-producing Escherichia Coli
TMA: Thrombotic Microangiopathy
TTP: Thrombotic Thrombocytopenic Purpura
UA: Urine analysis
WBC: White blood cell
