# Supplementary material for: Development and pilot implementation of Iranian Hemolytic Uremic Syndrome Registry
Source: Orphanet J Rare Dis. 2022 Jun 16;17:228. doi: 10.1186/s13023-022-02376-9 (PMC9205084; doi:10.1186/s13023-022-02376-9)
Supplement: Supplementary file 1 — Additional file 1: Interview questions. [file 13023_2022_2376_MOESM1_ESM.docx]

**Additional file 1**

**Interview questions**

1- What is the current process of identification, diagnosis and treatment of children with HUS in Iranian medical centers?

2. What centers are currently available for the treatment of HUS?

3- In the current process of diagnosis and treatment of patients with HUS what data are recorded and how is recorded? Electronically or manually?

4. How is HUS data currently stored in medical centers? What are the problems in the storage, quality, and access to this data?

5. What data sources are currently available for access to data on children with HUS in Iran? What are their problems? How is their quality of data?

6- Is it necessary to create a HUS registry in Iran?

7. In your opinion, what should be the purpose of setting up the HUS registry in Iran?

8- In your opinion, what are the inclusion criteria for patients in the Iranian HUS registry?

9. How do you think HUS cases should be identified?

10- In your opinion, what data sources can be used to collect Iranian HUS registry data?

11- In your opinion, what should be the method of data collection in the HUS registry of Iran?

12- In your opinion, what data should be recorded in the HUS registry of Iran?

13- In your opinion, what are the data quality control methods of the Iranian HUS registry?
