# Supplementary material for: Development and pilot implementation of Iranian Hemolytic Uremic Syndrome Registry
Source: Orphanet J Rare Dis. 2022 Jun 16;17:228. doi: 10.1186/s13023-022-02376-9 (PMC9205084; doi:10.1186/s13023-022-02376-9)
Supplement: Supplementary file 3 — Additional file 3: Results of the first round of Delphi technique regarding data classes and elements. [file 13023_2022_2376_MOESM3_ESM.docx]

**Additional file 3**

**Table 1 - Results of the first round of Delphi technique regarding data classes and elements**

| Data classes and elements | Number (percentage) of agreement (options 4 and 5) | Mean | Median | | Accepted/Rejected/ Delphi 2nd round |
| --- | --- | --- | --- | --- | --- |
| **Demographic** | | | | | |
| Name and family name | 23 (100) | 4.82 | | 5 | Accepted |
| Father's name | 21 (91) | 4.56 | | 5 | Accepted |
| ID Code | 23 (100) | 4.56 | | 5 | Accepted |
| Date of birth | 23 (100) | 4.82 | | 5 | Accepted |
| Age | 23 (100) | 4.86 | | 5 | Accepted |
| Gender | 23 (100) | 4.82 | | 5 | Accepted |
| Ethnic | 21 (91) | 4.56 | | 5 | Accepted |
| Address | 21 (91) | 4.56 | | 5 | Accepted |
| Place of birth | 21 (91) | 4.56 | | 5 | Accepted |
| Phone | 23 (100) | 4.73 | | 5 | Accepted |
| **Referral and hospitalization** | | | | | |
| Hospital name | 18 (78) | 4.08 | | 4 | Accepted |
| Patient referral physician | 4 (17) | 2.26 | | 2 | Rejected |
| Patient referral center (hospital, office, clinic) | 19 (83) | 4.13 | | 5 | Accepted |
| **Physical examinations** | | | | | |
| Observation of edema or dehydration | 23 (100) | 4.82 | | 5 | Accepted |
| Heart/lung sound auscultation | 23 (100) | 4.65 | | 5 | Accepted |
| Blood pressure | 23 (100) | 4.86 | | 5 | Accepted |
| Vital signs | 23 (100) | 4.65 | | 5 | Accepted |
| Height | 22 (96) | 4.69 | | 5 | Accepted |
| Weight | 22 (96) | 4.52 | | 5 | Accepted |
| BMI | 18 (78) | 4.13 | | 4 | Accepted |
| **Signs and symptoms** | | | | | |
| Diarrhea (bloody, non-bloody) | 23 (100) | 4.78 | | 5 | Accepted |
| Vomiting | 23 (100) | 4.69 | | 5 | Accepted |
| Nausea | 22 (96) | 4/69 | | 5 | Accepted |
| Abdominal pain | 23 (100) | 4.82 | | 5 | Accepted |
| Fever | 23 (100) | 4.73 | | 5 | Accepted |
| Paleness | 22 (96) | 4.52 | | 5 | Accepted |
| Fatigue | 21 (91) | 4.34 | | 5 | Accepted |
| Weakness and lethargy | 21 (91) | 4.43 | | 5 | Accepted |
| Headache | 23 (100) | 4.43 | | 4 | Accepted |
| Vertigo | 21 (91) | 4.43 | | 5 | Accepted |
| Increased blood pressure | 23 (100) | 4.69 | | 5 | Accepted |
| Urine discoloration (blood in the urine) | 23 (100) | 4.69 | | 5 | Accepted |
| Decreased urine volume | 23 (100) | 4.82 | | 5 | Accepted |
| Swelling in the limbs | 23 (100) | 4.65 | | 5 | Accepted |
| **Underlying disease and risk factors (causes)** | | | | | |
| Exposing to contaminated sources (such as contaminated water and food) | 23 (100) | 4.56 | | 5 | Accepted |
| Patient living area (villages, suburbs, etc.) | 23 (100) | 4.69 | | 5 | Accepted |
| Medications taking | 18 (78) | 4.13 | | 4 | Accepted |
| Exposing to radiotherapy or chemotherapy | 14 (61) | 3.56 | | 4 | Delphi 2nd round |
| Diet | 7 (30) | 3.69 | | 3 | Delphi 2nd round |
| Contact with livestock | 14 (61) | 4.13 | | 4 | Delphi 2nd round |
| Contact with agricultural pesticides | 11 (48) | 3.26 | | 3 | Delphi 2nd round |
| Scorpion bites (like Gadim scorpions) | 9 (39) | 3.65 | | 3 | Delphi 2nd round |
| Lack of complement components | 23 (100) | 4.86 | | 5 | Accepted |
| Underlying genetic diseases | 23 (100) | 4.78 | | 5 | Accepted |
| Cancer or malignancy | 18 (78) | 4.21 | | 4 | Accepted |
| Underlying collagen vascular diseases | 23 (100) | 4.65 | | 5 | Accepted |
| Underlying heart disease | 18 (78) | 4.21 | | 4 | Accepted |
| Underlying kidney disease | 23 (100) | 4.86 | | 5 | Accepted |
| Underlying high blood pressure | 23 (100) | 4.82 | | 5 | Accepted |
| Underlying infections | 20 (87) | 4.56 | | 5 | Accepted |
| Underlying systemic diseases | 21 (91) | 4.43 | | 5 | Accepted |
| **Laboratory Tests** | | | | | |
| Hematology | 23 (100) | 4.82 | | 5 | Accepted |
| Urine analysis | 23 (100) | 4.73 | | 5 | Accepted |
| Urine culture | 22 (96) | 4.69 | | 5 | Accepted |
| Stool culture | 22 (96) | 4.69 | | 5 | Accepted |
| Biochemistry | 23 (100) | 4.86 | | 5 | Accepted |
| Immunology | 21 (91) | 4.47 | | 5 | Accepted |
| Hemoglobin | 23 (100) | 4.86 | | 5 | Accepted |
| Platelet count | 23 (100) | 4.95 | | 5 | Accepted |
| Peripheral blood smear | 23 (100) | 4.95 | | 5 | Accepted |
| Creatinine and urea | 23 (100) | 4.95 | | 5 | Accepted |
| Reticulocytes | 23 (100) | 4.86 | | 5 | Accepted |
| Lactic dehydrogenase | 23 (100) | 4.82 | | 5 | Accepted |
| Calcium | 18 (78) | 4.26 | | 4 | Accepted |
| Sodium | 19 (83) | 4.43 | | 5 | Accepted |
| Potassium | 20 (87) | 4.56 | | 5 | Accepted |
| Phosphorus | 20 (87) | 4.63 | | 5 | Accepted |
| Urine output | 23 (100) | 4.86 | | 5 | Accepted |
| Presence of RBC and WBC in urine | 23 (100) | 4.86 | | 5 | Accepted |
| Cast in urine | 22 (96) | 4.65 | | 5 | Accepted |
| Coombs | 21 (91) | 4.65 | | 5 | Accepted |
| Factors related to the complement system such as C4, C3, CFB, CFI, CFH | 23 (100) | 4.91 | | 5 | Accepted |
| ADAMTS13 | 23 (100) | 4.86 | | 5 | Accepted |
| ANA | 23 (100) | 4.82 | | 5 | Accepted |
| Albumin protein | 23 (100) | 4.78 | | 5 | Accepted |
| Total protein | 20 (87) | 4.36 | | 5 | Accepted |
| Genetic test result and upload report | 23 (100) | 4.86 | | 5 | Accepted |
| **Disease history** | | | | | |
| Past history | 23 (100) | 4.82 | | 5 | Accepted |
| Family history (sibling | 23 (100) | 4.86 | | 5 | Accepted |
| Age at onset of symptoms | 23 (100) | 4.78 | | 5 | Accepted |
| Number of days of onset of symptoms at admission | 23 (100) | 4.78 | | 5 | Accepted |
| Duration of diagnosis until patient registration | 22 (96) | 4.65 | | 5 | Accepted |
| History of gastrointestinal infection (especially Shiga-toxin) in the previous two weeks (dysentery) | 23 (100) | 4.82 | | 5 | Accepted |
| History of respiratory infection two weeks ago (cold or acute pneumonia) | 23 (100) | 4.69 | | 5 | Accepted |
| History of systemic diseases | 20 (87) | 4.47 | | 5 | Accepted |
| History of kidney transplantation | 19 (83) | 4.39 | | 5 | Accepted |
| Parental consanguineous marriage | 17 (74) | 4.13 | | 4 | Delphi 2nd round |
| **Paraclinical measures** | | | | | |
| Abdominal ultrasound and its outcome | 23 (100) | 4.69 | | 5 | Accepted |
| Echocardiography and its outcome | 22 (96) | 4.69 | | 5 | Accepted |
| MRI and its result | 18 (78) | 4.39 | | 5 | Accepted |
| Chest photo and its result | 22 (96) | 4.69 | | 5 | Accepted |
| Brain CT scan and its result | 21 (91) | 4.56 | | 5 | Accepted |
| **Treatments and medications** | | | | | |
| Supportive therapies | 23 (100) | 4.78 | | 5 | Accepted |
| Antibiotics | 22 (96) | 4.69 | | 5 | Accepted |
| Lowering blood pressure like Enalapril | 23 (100) | 4.82 | | 5 | Accepted |
| Anticoagulants | 20 (87) | 4.56 | | 5 | Accepted |
| Coagulation drugs | 23 (100) | 4.65 | | 5 | Accepted |
| Eculizumab | 23 (100) | 4.78 | | 5 | Accepted |
| Rituximab | 20 (87) | 4.60 | | 5 | Accepted |
| Immunoglobulins | 21 (91) | 4.52 | | 5 | Accepted |
| Steroids | 22 (96) | 4.65 | | 5 | Accepted |
| Immunosuppressive drugs | 23 (100) | 4.65 | | 5 | Accepted |
| Pulse methylprednisolone | 23 (100) | 4.60 | | 5 | Accepted |
| Monoclonal antibodies | 23 (100) | 4.69 | | 5 | Accepted |
| Dosage of drugs | 18 (78) | 4.26 | | 5 | Accepted |
| Duration of medication | 18 (78) | 4.26 | | 5 | Accepted |
| Date of consumption of the first dose | 21 (91) | 4.52 | | 5 | Accepted |
| Plasma injection | 23 (100) | 4.82 | | 5 | Accepted |
| Plasma exchange | 23 (100) | 4.82 | | 5 | Accepted |
| Date of first treatment | 11 (48) | 3.56 | | 3 | Delphi 2nd round |
| Hemodialysis or peritoneal dialysis (with results) | 23 (100) | 4.69 | | 5 | Accepted |
| Kidney transplant (with results) | 23 (100) | 4.69 | | 5 | Accepted |
| Splenectomy (with results) | 20 (87) | 4.52 | | 5 | Accepted |
| Liver transplantation (with results) | 20 (87) | 4.43 | | 5 | Accepted |
| **Complications and Outcomes** | | | | | |
| Recurrence of the disease | 23 (100) | 4.73 | | 5 | Accepted |
| Severe kidney failure | 23 (100) | 4.73 | | 5 | Accepted |
| Severe anemia | 23 (100) | 4.69 | | 5 | Accepted |
| Neurological complications such as seizures | 23 (100) | 4.82 | | 5 | Accepted |
| Diabetes | 23 (100) | 4.86 | | 5 | Accepted |
| Hypertension | 23 (100) | 4.91 | | 5 | Accepted |
| Increased blood protein levels in the long term | 23 (100) | 4.78 | | 5 | Accepted |
| Side effects of medications and treatments (during hospitalization) | 23 (100) | 4.69 | | 5 | Accepted |
| Severity of complications and consequences | 23 (100) | 4.82 | | 5 | Accepted |
| Depression | 21 (91) | 4.56 | | 5 | Accepted |
| Systemic lupus erythematosus | 8 (35) | 3.34 | | 3 | Delphi 2nd round |
| Bleeding | 23 (100) | 4.78 | | 5 | Accepted |
| Sepsis | 23 (100) | 4.86 | | 5 | Accepted |
| Meningococcal infections | 17 (74) | 4.13 | | 4 | Delphi 2nd round |
| Other serious infections | 23 (100) | 4.73 | | 5 | Accepted |
| Death (main date and cause of death) | 23 (100) | 4.91 | | 5 | Accepted |
| **Patient status at discharge** | | | | | |
| Recovery | 23 (100) | 4.78 | | 5 | Accepted |
| Discharge along with renal failure | 23 (100) | 4.65 | | 5 | Accepted |
| Recommend to follow | 23 (100) | 4.69 | | 5 | Accepted |
| Discharges Against Medical Advice (AMA) | 16 (70) | 3.91 | | 4 | Delphi 2nd round |
| **Patient follow-up** | | | | | |
| Clinical signs | 23 (100) | 4.95 | | 5 | Accepted |
| Laboratory findings | 23 (100) | 4.86 | | 5 | Accepted |
| Medications used | 23 (100) | 4.86 | | 5 | Accepted |
| Paraclinical procedures performed | 23 (100) | 4.82 | | 5 | Accepted |
