# Supplementary material for: Development and pilot implementation of Iranian Hemolytic Uremic Syndrome Registry
Source: Orphanet J Rare Dis. 2022 Jun 16;17:228. doi: 10.1186/s13023-022-02376-9 (PMC9205084; doi:10.1186/s13023-022-02376-9)
Supplement: Supplementary file 4 — Additional file 4: Results of the software usability evaluation. [file 13023_2022_2376_MOESM4_ESM.docx]

**Additional file 4**

**Table 1 - Results of the software usability evaluation**

| QUIS dimension | Sub-demotions | Sub-dimension  Score±(SD) | Dimension score ±SD |
| --- | --- | --- | --- |
| **Overall reactions to the software** | Overall software performance | 7.7±0.50 | 8±0.57 |
|  | Easy to use | 9±0 |  |
|  | Feeling comfortable in using system | 8.5±0.57 |  |
|  | Overall system design | 8±0.81 |  |
|  | Continuous use of the system | 7.7±0.95 |  |
|  | System settings | 7.5±0.57 |  |
| **Screen** | Characters on the computer screen | 8.7±0.50 | 8.2±0.29 |
|  | Highlighting on the screen simplifies task | 8.2±0.95 |  |
|  | Organization of information on screen | 8.2±0.50 |  |
|  | Sequence of screens | 8±0.81 |  |
| **Terminology/system information** | Use of terms throughout system | 9±0 | 8.3±0.38 |
|  | Computer terminology is related to the task you are doing | 8.2±0.95 |  |
|  | Position of messages on screen | 8.2±0.95 |  |
|  | Messages on screen which prompt user for input | 8.7±0.50 |  |
|  | Computer keeps you informed about what it is doing | 8±0.81 |  |
|  | Error messages | 8.2±0.95 |  |
| **Learning** | Learning to operate the system | 8.5±0.57 | 8.1±0.61 |
|  | Exploring new features by trial and error | 8.2±0.95 |  |
|  | Remembering names and use of commands | 8.5±0.57 |  |
|  | Tasks can be performed in a straight-forward manner | 8.2±0.95 |  |
|  | Help messages on the screen | 8.7±0.50 |  |
|  | Supplemental reference materials | 7±0.81 |  |
| **System capabilities** | System speed | 8±0.81 | 7.91±0.17 |
|  | System reliability | 8.2±0.95 |  |
|  | System tends to be | 7.7±0.95 |  |
|  | Correcting your mistakes | 8±0.81 |  |
|  | Designed for all levels of users | 8±0.81 |  |
